# Fixed Drug Eruption on the Penis Due to Trimethoprim-Sulfamethoxazole: A Case Report

**DOI:** 10.7759/cureus.65422

**Published:** 2024-07-26

**Authors:** Andres F Shapiro, Leticia M Garcia Borbor

**Affiliations:** 1 Department of Health, Fundación Jesús Carpintero, La Libertad, ECU

**Keywords:** fixed drug eruption, post-inflammatory hyperpigmentation, penis, trimethoprim-sulfamethoxazole, clinical case report

## Abstract

A fixed drug eruption (FDE) is a common dermatological drug side effect but can go unnoticed. It is characterized by an oval-circular erythematous patch, sometimes with itching and burning pain localized in many parts of the body, such as the face, lips, torso, limbs, and anogenital area. Its diagnosis is generally clinical, but it can be mistaken for other dermatological diseases seen in primary care, like balanitis, genital herpes, and lichen planus. It can be a diagnostic challenge for primary care physicians when it is not considered. We present a 26-year-old man who developed an FDE in the penis with intense itching and burning pain during his labor hours after 15 minutes of consuming an oral dose of trimethoprim-sulfamethoxazole for a gastrointestinal infection. The patient was treated with topical corticosteroids twice per day and educated to avoid the use of the antibiotic again. In the next few days, the symptoms fully resolved, and he developed post-inflammatory hyperpigmentation in the area. The primary management of an FDE is immediate discontinuation of the offending drug and use of topical corticosteroids to prevent possible generalized reactions and recurrence of lesions. Therefore, the primary care physician should consider this condition in his or her diagnosis when new dermatologic lesions occur after exposure to a new drug.

## Introduction

A fixed drug eruption (FDE) is one of the most common dermatological drug side effects. It can manifest as an oval-circular erythematous patch with or without symptoms like pain and itching in many parts of the body like the face, torso, limbs, and genitalia including mucous membranes, but its clinical manifestation depends on the causative drug [1]. Delaying the withdrawal of the offending drug increases the symptoms and distress of the patient, including the possibility of a general reaction. Therefore, it is important to take in mind that an FDE is an alternative diagnosis in front of other penile lesions seen in primary care as balanitis, genital herpes, and lichen planus [2].

More than 100 drugs have been reported to cause FDEs with a prevalence of 14-22% in adults and children [3,4]. Trimethoprim-sulfamethoxazole is one of the most common drugs used in primary care to treat many types of infections and has also been reported as one of the most causative drugs of FDEs with a maximum incidence of 36.3% in a case series of 113 patients where the lips were the site most affected, followed by the trunk and genitalia [5]. Here, we report a case of an FDE of trimethoprim-sulfamethoxazole on the penis.

## Case presentation

**Figure 1:**
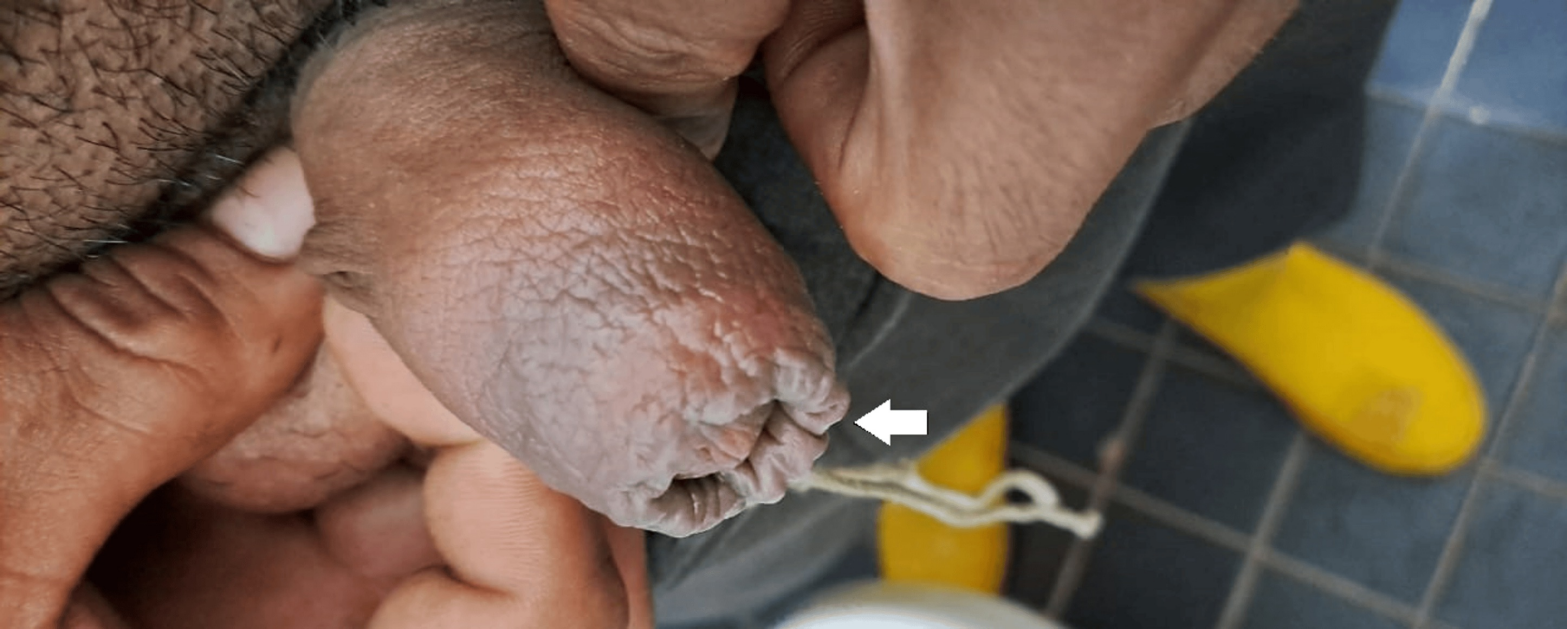
Edematous foreskin with erythema of the penis. There was a difficult and painful retraction of the foreskin.

We present a 26-year-old aquaculture worker and Ecuadorian man without any relevant past medical backgrounds including sexually transmissible disease, with a stable sexual partner, who, during working hours, complained of intensive itching and mild burning pain in the penis after 15 minutes of taking a dose of trimethoprim-sulfamethoxazole 80 mg/400 mg for a gastrointestinal infection diagnosed in the morning of the same day. During the evaluation, he denied traumas or exposition to the chemical used in his workplace in his genital area, and there were no urinary or systemic symptoms. Physical examination revealed an erythematous lesion with moderate edema of the foreskin with painful retraction (Figure 1), and there was no evidence of other lesions in the body.

Because of the clinical history and the relationship interval between the administration of the drug and the appearance of the symptoms, an FDE seems to be the presumptive diagnosis. A differential diagnosis taken into account was allergic contact dermatitis produced by many of the chemical products used in his laboral charge, which was a prevalent condition in the workplace. We were not available to access laboratory tests because of the difficulty accessing health services localized in a rural area, so we proceeded to withdraw the drug and prescribed a betamethasone 0.05% cream twice daily for five days. We also taught the patient about FDE and its relationship with the drug used and the importance of avoiding new expositions.

**Figure 2:**
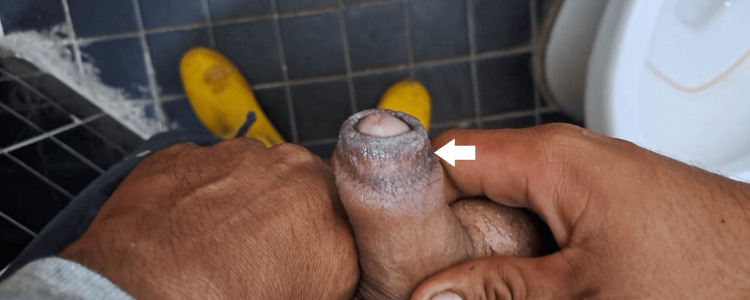
Postinflammatory hyperpigmentation of the foreskin one week later.

In the further days, the patient reported that the symptoms ceased, and one week later, we found no edema, but just a hyperpigmentation of the foreskin as a sequel (Figure 2).

Despite the effort to teach the patient his FDE to trimethoprim-sulfamethoxazole, the patient suffered again a new exposure to the drug in the next month, developing in approximately 10 minutes the same clinical symptoms mentioned before in the same area. To this, we had to repeat the same treatment and reinforce patient education about the condition. During the next follow-up by the occupational doctor of the workplace, there was no report of new episodes of FDE in the patient.

## Discussion

An FDE is one of the common dermatological side effects characterized by oval-circular lesions as erythematous patches generally without systemic symptoms [1]. This reaction could develop in several parts of the body, such as the face, lips, trunk, and limbs including the anogenital area due to immunological reactions classified as type IV hypersensitivity front several classes of drugs, such as nonsteroidal anti-inflammatory drugs and antibiotics [6].

The proposed pathogenesis of an FDE begins with activation of intraepidermal CD8+ T cells by interferon-gamma (IFN-γ) and local mast cells; once activated, CD8+ T cells begin keratinocyte destruction in conjunction with subsequently recruited CD4+ T cells. At the end of the process, the number of CD8+ T cells decreases, remaining as effector memory T cells [7]. This pathogenic model only explains the local lesions in an FDE that are auto-limited and recover in weeks, being able to leave post-inflammatory hyperpigmentation; however, there are recurrences before the exposition to the causative drug [6,7].

Many of these lesions can be a diagnostic challenge by having a similar morphology to other dermatological diseases, including genital lesions. Hence, our case report is a clear example that FDEs should always be taken into account in medical general practice because the avoidance of a causative drug is a key step for the recovery of the patient. However, the limitation of our case report was the incapacity to approach the patient with diagnosis tests, like patch testing, lymphocyte transformation test, or oral challenge (which nowadays is not recommended owing to the risk of producing a generalized FDE [7] ) due to the lack of access. Nevertheless, the diagnosis confirmation could be obtained due to the development of the same clinical picture to the patient's re-exposition to the drug, but this event highlights the efforts that practitioners have to make with patient education.

## Conclusions

FDEs can be caused by many types of drugs, such as trimethoprim-sulfamethoxazole, and manifest in different parts of the body with many morphologic variants, which could present a diagnostic challenge to the general practitioner if not taken into account. An FDE in the penis is very stressful for a patient, so an early diagnosis through the association of new drug exposure and the appearance of the lesion must be considered in the clinical history in general practice. Early withdrawal of the offending drug and the use of topical corticosteroids is the mainstay of treatment, but a key step is patient education about the condition and reinforcement at every opportunity to avoid new re-exposure.
